# Mother, Infant, and Household Factors Associated with the Type of Food Infants Receive in Developing Countries

**DOI:** 10.3389/fped.2014.00014

**Published:** 2014-02-28

**Authors:** Benjamin Yarnoff, Benjamin Allaire, Patrick Detzel

**Affiliations:** ^1^RTI International, Research Triangle Park, NC, USA; ^2^Nestlé Research Center, Lausanne, Switzerland

**Keywords:** infant feeding practices, determinants of feeding, developing countries, nutrition, infant feeding policy

## Abstract

**Objectives:** We explore the complex factors associated with infant feeding by analyzing what mother, infant, and household factors are associated with the types of food given to infants. We seek to quantify associations in order to inform public health policy about the importance of target populations for infant feeding programs.

**Methods:** We used data from the Demographic Health Survey in 20 developing countries for multiple years to examine mother, infant, and household factors associated with six types of food given to infants (exclusive breastfeeding, non-exclusive breastfeeding, infant formula, milk liquids, non-milk liquids, and solid foods). We performed a seemingly unrelated regressions analysis with community-year fixed effects to account for correlation between food types and control for confounding factors associated with community resources, culture, time period, and geography in the pooled analysis.

**Results:** We found that several mother, infant, and household characteristics were associated with each of the feeding types. Most notably, mother’s education, working status, and weight are significantly associated with the type of food given to infants. We provide quantified estimates of the association of each of these variables with six types of food given to infants.

**Conclusion:** By identifying maternal characteristics associated with infant feeding and quantifying those associations, we help public health policymakers generate priorities for targeting infant feeding programs to specific populations that are at greatest risk. Higher educated, working mothers are best to target with exclusive breastfeeding programs for young infants. Mothers with lower education are best to target with complementary feeding programs in infants older than 1 year. Finally, while maternal weight is associated with higher levels of exclusive breastfeeding the association is too weak to merit targeting of breastfeeding programs to low-weight mothers.

## Introduction

Current recommendations by the World Health Organization (WHO) and UNICEF state that mothers should exclusively breastfeed infants for the first 6 months of life and should provide complementary foods and sustained breastfeeding up to 2 years of age or beyond (1). Despite these recommendations, recent evidence suggests that in developing countries as many as 22% of mothers may feed their infants solid foods prior to 6 months of age (2, 3). Even if solid foods are introduced at the proper time, mothers may either select foods lacking essential nutrients or not provide enough complementary foods to make up a nutrient deficit (4).

Preliminary research has tried to identify potential explanations for this phenomenon, but typically relies on smaller, country-specific samples and a limited set of explanatory variables. A survey of mothers in Botswana found that the predominant reason for breastfeeding cessation was that the mother was at work or at school (5). A survey of women in the north of Tehran found that early breastfeeding cessation was associated with low birth weight and Cesarean delivery (6). A survey in Lebanon found that exclusive breastfeeding was associated with delivery type and place of residence (urban/rural) and was inversely related to the mother’s education (7). One past study examined a continuum of feeding types for infants older than 6 months and found differential feeding patterns by maternal education. Their sample consisted of several countries located in Latin America (8). While not exhaustive, these examples give an idea of the nature of past research in this area.

Our study builds on this past work to expand understanding of infant feeding patterns. We provide several important advances over past work. First, we explicitly examine factors associated with six different food types rather than simply exclusive breastfeeding. This is important because different non-breast-milk food types have been found to have different effects on infant health (3). Second, we use a large and geographically diverse dataset that enables us to produce results with greater external generalizability. Third, we used methods that control for confounding factors by using community-year fixed effects and control for correlation between the feeding types by using a seemingly unrelated regressions (SUR) technique. Finally, we quantify the association between mother, infant, and household factors and feeding practices.

This method of analysis using community-year fixed effects to control for geography and time (and all other unobserved factors associated with those two such as social and cultural setting) in effect compares infants within the same geographic settings and time periods to determine associations between feeding and mother, infant, and household factors within a community in a given year and then averages these inter-community estimates to produce a pooled average estimate. Generalized population averages such as this are necessary when examining population-level policies, because they provide policymakers across a broad spectrum with average associations between household, maternal, and child factors and infant feeding practices. For example, when considering a breastfeeding program targeted at working mothers, a policymaker may be interested in answering questions such as “on average how much less likely are working mothers to breastfeed their infants?” The estimates generated in this research provide policymakers with quantified values that can be used to answer these questions for policy planning.

## Materials and Methods

We compared mother, infant, and household characteristics of infants receiving each of six different food types and used seemingly unrelated regression analysis with community-year fixed effects (discussed in more detail below) to identify factors associated with an infant receiving each food type. This research was approved by the Institutional Review Board (IRB) and conducted in accord with all prevailing ethical principles of public health research.

### Data sources

We used publicly available data from the Demographic Health Survey (DHS) for multiple survey years from 20 developing countries (see Table A1 in Appendix for the full list of countries and years). We decided *ex ante* to examine 20 countries from the Africa, Asia, and Latin America regions, and selected these countries based on the following criteria: multiple survey years per country and the presence of information on infant feeding and maternal characteristics in each survey. Countries were selected to maximize these two criteria while also maintaining a distribution across the regions. We tested whether this subsample was representative of the full sample of DHS surveys by comparing mean measures of breastfeeding for infants age 0–6 months, under-5 mortality, and diarrhea incidence between our subsample and the full sample. Mean indicators were virtually identical between this subsample and the full sample of DHS surveys for exclusive breastfeeding among infants 0–6 months old (35% in the full sample and 33% in the subsample), non-exclusive breastfeeding among infants 0–6 months old (61% in the full sample and 63% in the subsample), under-5 mortality in the past 5 years (99 per 1000 in the full sample and 101 per 1000 in the subsample), and diarrhea incidence (20% in the full sample and 21% in the subsample). These similarities suggest that this subsample is representative of overall feeding practices in the full DHS sample and that results are generalizable across the full geography of the DHS. We also tested the effect that older surveys have on the external generalizability of results by conducting the analysis on only a sample of recent surveys. The results from this subsample analysis (not reported) were very similar to those from the full sample, which suggests that the older surveys do not jeopardize external generalizability of the results.

The DHS is a nationally representative survey of women aged 15–49 that is funded by USAID, receives technical support from the US Census Bureau, and is conducted in developing countries approximately every 5 years. The survey asks about the health of each woman and her infants as well as demographics and socioeconomics. Importantly, the DHS asks about all foods that the woman’s youngest infant was given in the past 24 h. Our sample consists of 116,700 infants divided across three age groups: 0–6 months (34,290 infants), 6 months to 1 year (29,630 infants), and 1–2 years (52,780 infants).

### Variables

The dependent variables of interest are the types of food an infant was fed in the past 24 h. We took data from the 24-h infant food recall to create dummy variables for six types of food: exclusive breastfeeding, non-exclusive breastfeeding, infant formula, milk liquids, non-milk liquids, and solid foods. The food recall variables that went into each food type definition are provided in Table A2 in Appendix. Each of these indicator variables is equal to one if the infant received that type of food in the past 24 h and zero otherwise. An infant was categorized as being exclusively breastfed if he or she was breastfed in the past 24 h, but did not receive any other types of food. This construction measures only whether the infant was exclusively breastfed in the past 24 h rather than on a regular basis. Note that the food types other than exclusive breastfeeding are not mutually exclusive so that an infant may receive, for example, both infant formula and milk liquids or both non-exclusive breastfeeding and solid foods.

Independent variables of interest are mother, infant, and household factors that may influence the type of food to give their infants. We examined the following variables: mother’s education (indicator variables for no formal education, incomplete primary, complete primary, incomplete secondary, complete secondary, and post-secondary), mother’s work status (indicator variable for whether she works outside the home), mother’s occupation (indicator variables for no occupation and nine occupation categories), mother’s weight (in kilograms), mother’s height (in centimeters), relative infant size at birth as reported by the mother (indicator variables for very small, smaller than average, average, larger than average, and very large), whether the household has agricultural land, number of household members, number of infants in the household, infant gender, and infant vaccination history (dummy variable for whether the infant has received any vaccination).

### Statistical methodology

To account for interrelationships between the six food types, we used a SUR methodology to estimate the effect of characteristics on determining the food types given to an infant in the past 24 h. SUR estimates a system of six linear equations (one for each food type) where the error terms of each equation are related. This regression methodology was employed to account for overlap across each food type and components of the error term that could be common across the different food types.

A concern with the estimation of household decisions is that community resources and geographic factors that vary from year to year will confound estimates of the effect of mother, infant, and household characteristics on the type of food an infant receives. Our dataset represents a diverse set of countries and time periods, making confounding an especially salient concern. Thus, to control for these confounding factors, we included community-year fixed effects. The fixed effects control for all community factors in a given year, including availability of health resources and types of food, geographical disease, and agriculture characteristics, and weather history. For example, a mother living in a community that experiences drought in given year may be less likely to feed her baby corn. Because, these factors are likely to bias estimates by influencing the availability of food type, these fixed effects are essential to producing unbiased estimates. Communities are defined by the DHS primary sampling unit for each survey year. An analysis with community-year fixed effects can be thought of as comparing infants within the same geographic settings and time period to determine associations between feeding and mother, infant, and household factors within that community and then averaging these inter-community estimates to produce a pooled average estimate.

The independent variables described above also serve as controls for potentially confounding factors. Most important among them is infant size at birth which represents the infant’s genetic health endowment and all health inputs given to an infant *in utero* such as maternal nutrition and care (9, 10) and thus is a proxy for unobserved household health behaviors and resources.

We performed our analysis separately for three age groups: less than 6 months old, 6 months to 1-year-old, and 1–2 years old. We used Stata (version 11.2) to perform all statistical analysis. We weighted all analyses using DHS sample weights.

## Results

Table 1 presents means for the six food types as well as mother, infant, and household characteristics, by age group. Notably, infants in all three age groups are fed a wide range of foods. The samples consist of primarily low educated mothers (approximately 75% have primary education or less). Approximately half of the mothers work outside the home, largely in sales and agriculture. Finally of note, mothers in the sample are generally smaller in height and weight than in developed countries such as the United States (11).

**Table 1:** **Summary statistics, by age group**.

Variable	Age <6 months	Age 6 months to 1 year	Age 1–2 years
**BREASTFEEDING**			
Exclusive breastfeeding	0.33	0.04	0.03
Non-exclusive breastfeeding	0.63	0.87	0.73
**FOOD TYPE IF NON-EXCLUSIVELY BREASTFEEDING**			
Infant formula	0.19	0.14	0.11
Milk liquids	0.27	0.33	0.37
Non-milk liquids	0.88	0.95	0.97
Solid foods	0.39	0.82	0.91
**FOOD TYPE IF NO BREASTFEEDING**			
Exclusive infant formula	0.04	0.00	0.00
Non-exclusive infant formula	0.60	0.55	0.26
Milk liquids	0.54	0.69	0.58
Non-milk liquids	0.85	0.96	0.97
Solid foods	0.60	0.94	0.98
**MOTHER’S EDUCATION**			
No formal education	0.37	0.35	0.36
Incomplete primary	0.23	0.24	0.24
Complete primary	0.12	0.12	0.11
Incomplete secondary	0.16	0.16	0.16
Complete secondary	0.08	0.08	0.08
Post-secondary	0.04	0.05	0.04
Mother is currently employed outside the home	0.47	0.52	0.54
**MOTHER’S OCCUPATION**			
No occupation	0.49	0.45	0.43
Professional, technical, manager	0.03	0.03	0.03
Clerical	0.01	0.01	0.01
Sales	0.11	0.11	0.11
Agriculture – self-employed	0.24	0.26	0.26
Agriculture – employe	0.05	0.05	0.06
Domestic services	0.01	0.01	0.01
Services	0.02	0.02	0.03
Skilled manual	0.04	0.04	0.04
Unskilled manual	0.02	0.02	0.02
**BIRTH SIZE**			
Very large	0.05	0.05	0.05
Larger than average	0.19	0.20	0.20
Average	0.57	0.56	0.56
Smaller than average	0.14	0.13	0.13
Very small	0.05	0.05	0.05
Mother’s height (cm)	156	156	156
Mother’s weight (kg)	55	55	55
Total in household	7.05	6.87	6.76
Children in household	2.12	2.04	1.93
Agricultural household	0.14	0.16	0.16
Female infant	0.50	0.49	0.49
Infant ever vaccinated	0.74	0.91	0.93

We find several factors are associated with whether certain food types are given to infants under the age of 6 months (Table 2). Maternal education is significantly correlated with all food types. The correlation does not have a consistent sign for breastfeeding, non-milk liquids, and solid foods, but is positive for infant formula and milk liquids. Infants with mothers that are currently working are less likely to be exclusively breastfed and more likely to be non-exclusively breastfed and to receive milk liquids, non-milk liquids, and solid foods. Maternal work status has no link to the use of infant formula. Mother’s weight is associated with increases in the use of exclusive breastfeeding, infant formula, and non-milk liquids and decreases in the use of non-exclusive breastfeeding, non-milk liquids, and solid foods.

**Table 2:** **Associations with feeding type, age <6 months**.

Variable	Exclusive breastfeeding	Non-exclusive breastfeeding	Infant formula	Milk liquids	Non-milk liquids	Solid foods
**MOTHER’S EDUCATION (BASE CASE *=* NO FORMAL EDUCATION)**						
Incomplete primary	0.001	−0.002	0.004	0.013**	0.007	0.006
	(0.007)	(0.007)	(0.004)	(0.005)	(0.007)	(0.006)
Complete primary	0.016*	−0.017*	0.023**	0.033**	−0.010	−0.037**
	(0.009)	(0.009)	(0.005)	(0.007)	(0.009)	(0.008)
Incomplete secondary	−0.010	0.007	0.041**	0.037**	0.001	−0.009
	(0.009)	(0.009)	(0.005)	(0.007)	(0.009)	(0.008)
Complete secondary	−0.036**	0.030**	0.071**	0.067**	0.014	0.020*
	(0.012)	(0.013)	(0.006)	(0.009)	(0.013)	(0.011)
Post-secondary	0.014	−0.029	0.112**	0.062**	−0.062**	−0.029*
	(0.018)	(0.018)	(0.009)	(0.014)	(0.018)	(0.016)
Mother is currently employed outside the home	−0.073**	0.079**	0.007	0.033**	0.072**	0.081**
	(0.011)	(0.011)	(0.006)	(0.008)	(0.011)	(0.010)
Mother works at home	−0.020**	0.017**	0.004	−0.000	0.018**	0.010
	(0.008)	(0.008)	(0.004)	(0.006)	(0.008)	(0.007)
**MOTHER’S OCCUPATION (BASE CASE ***=*** NO OCCUPATION)**						
Professional, technical, manager	0.051**	−0.053**	0.043**	0.014	−0.039	−0.031
	(0.025)	(0.025)	(0.013)	(0.019)	(0.025)	(0.022)
Clerical	0.027	−0.046	0.049**	0.074**	−0.051	−0.006
	(0.033)	(0.034)	(0.017)	(0.025)	(0.034)	(0.030)
Sales	0.042**	−0.045**	0.008	−0.023*	−0.054**	−0.064**
	(0.018)	(0.018)	(0.009)	(0.014)	(0.018)	(0.016)
Agriculture – self-employed	0.053**	−0.050**	−0.012	−0.025*	−0.044**	−0.056**
	(0.019)	(0.019)	(0.010)	(0.014)	(0.019)	(0.017)
Agriculture – employe	0.031	−0.013	0.000	−0.016	−0.031	−0.040**
	(0.021)	(0.021)	(0.011)	(0.016)	(0.021)	(0.019)
Domestic services	0.057	−0.024	0.030*	0.017	−0.110**	−0.046
	(0.035)	(0.035)	(0.018)	(0.026)	(0.035)	(0.032)
Services	0.039	−0.035	−0.014	−0.010	−0.034	−0.065**
	(0.025)	(0.025)	(0.013)	(0.019)	(0.025)	(0.023)
Skilled manual	0.044**	−0.049**	0.007	−0.031**	−0.042**	−0.057**
	(0.019)	(0.019)	(0.010)	(0.014)	(0.019)	(0.017)
Unskilled manual	−0.004	−0.007	−0.006	−0.038**	0.009	−0.048**
	(0.025)	(0.025)	(0.013)	(0.019)	(0.025)	(0.023)
**BIRTH SIZE (BASE CASE ***=*** VERY LARGE)**						
Larger than average	−0.003	0.003	−0.001	−0.002	−0.002	−0.013
	(0.012)	(0.012)	(0.006)	(0.009)	(0.012)	(0.011)
Average	0.002	−0.003	0.003	−0.004	−0.005	−0.007
	(0.011)	(0.011)	(0.006)	(0.008)	(0.011)	(0.010)
Smaller than average	0.002	−0.006	−0.007	0.023**	−0.021*	−0.025**
	(0.012)	(0.012)	(0.006)	(0.009)	(0.012)	(0.011)
Very small	−0.022	0.014	0.009	0.025**	0.005	−0.009
	(0.015)	(0.015)	(0.008)	(0.011)	(0.015)	(0.013)
Mother’s height	−0.000	0.000	−0.001**	−0.000	0.000	0.000
	(0.000)	(0.000)	(0.000)	(0.000)	(0.000)	(0.000)
Mother’s weight	0.001**	−0.001**	0.001**	0.000**	−0.001**	−0.001**
	(0.000)	(0.000)	(0.000)	(0.000)	(0.000)	(0.000)
Agricultural household	0.002	−0.006	0.003	−0.010	0.002	0.017**
	(0.009)	(0.009)	(0.005)	(0.007)	(0.009)	(0.008)
Total in household	−0.001	0.001	0.001**	0.001	−0.001	0.001
	(0.001)	(0.001)	(0.000)	(0.001)	(0.001)	(0.001)
Children in household	0.011**	−0.010**	−0.004**	0.001	−0.008**	−0.012**
	(0.002)	(0.003)	(0.001)	(0.002)	(0.003)	(0.002)
Female	−0.008*	0.008*	0.005**	0.003	0.012**	0.008**
	(0.005)	(0.005)	(0.002)	(0.003)	(0.005)	(0.004)
Ever vaccinated	−0.190**	0.189**	0.019**	0.077**	0.166**	0.174**
	(0.006)	(0.006)	(0.003)	(0.004)	(0.006)	(0.005)
Observations	34,290	34,290	34,290	34,290	34,290	34,290

*Standard errors (clustered on community-year) are given in parentheses*.

*All regressions include community-year fixed effects*.

*p < 0.10; **p < 0.05.

Table 3 contains results for the analysis of infants between the ages of 6 months and 1 year. The coefficients on maternal education are again mixed in sign for breastfeeding, but are positive and statistically significant for infant formula, milk liquids, non-milk liquids, and solid foods. Maternal work status is correlated with increases in the use of infant formula but has no association with other food types. Infants that were smaller at birth are more likely to be exclusively breastfed. Increasing mother’s weight is associated with decreases in non-exclusive breastfeeding and increases in the use of infant formula, milk liquids, and non-milk liquids.

**Table 3:** **Associations with feeding type, age 6 months to 1 year**.

Variable	Exclusive breastfeeding	Non-exclusive breastfeeding	Infant formula	Milk liquids	Non-milk liquids	Solid foods
**MOTHER’S EDUCATION (BASE CASE *=* NO FORMAL EDUCATION)**						
Incomplete primary	−0.001	0.005	0.005	0.021**	−0.002	0.022**
	(0.003)	(0.005)	(0.005)	(0.007)	(0.005)	(0.006)
Complete primary	−0.006	0.009	0.014**	0.044**	−0.001	0.031**
	(0.004)	(0.006)	(0.006)	(0.009)	(0.006)	(0.008)
Incomplete secondary	−0.017**	0.012**	0.051**	0.103**	0.020**	0.044**
	(0.004)	(0.006)	(0.006)	(0.008)	(0.006)	(0.008)
Complete secondary	−0.030**	0.013	0.090**	0.112**	0.032**	0.100**
	(0.006)	(0.008)	(0.008)	(0.012)	(0.009)	(0.011)
Post-secondary	−0.033**	0.021*	0.181**	0.115**	0.038**	0.120**
	(0.008)	(0.011)	(0.011)	(0.017)	(0.012)	(0.015)
Mother is currently employed outside the home	−0.009	0.009	0.031**	0.003	−0.009	0.002
	(0.006)	(0.009)	(0.009)	(0.013)	(0.009)	(0.012)
Mother works at home	0.004	−0.004	0.004	−0.012	−0.006	0.021**
	(0.003)	(0.005)	(0.005)	(0.007)	(0.005)	(0.007)
**MOTHER’S OCCUPATION (BASE CASE ***=*** NO OCCUPATION)**						
Professional, technical, manager	0.004	−0.033**	0.045**	0.119**	0.024	−0.050**
	(0.012)	(0.017)	(0.016)	(0.025)	(0.017)	(0.023)
Clerical	−0.001	−0.038*	0.069**	0.176**	0.053**	−0.050*
	(0.015)	(0.021)	(0.021)	(0.032)	(0.022)	(0.029)
Sales	−0.002	0.019	−0.059**	0.016	0.021	−0.007
	(0.009)	(0.012)	(0.012)	(0.019)	(0.013)	(0.017)
Agriculture – self – employed	−0.003	0.010	−0.044**	−0.007	0.038**	−0.020
	(0.009)	(0.013)	(0.013)	(0.019)	(0.013)	(0.018)
Agriculture – employe	0.003	0.007	−0.030**	0.002	0.032**	−0.005
	(0.010)	(0.014)	(0.014)	(0.021)	(0.015)	(0.019)
Domestic services	−0.008	−0.020	−0.063**	0.078**	0.014	−0.009
	(0.016)	(0.022)	(0.022)	(0.033)	(0.023)	(0.030)
Services	−0.015	0.012	−0.032*	0.084**	0.031*	−0.034
	(0.012)	(0.016)	(0.016)	(0.025)	(0.017)	(0.022)
Skilled manual	−0.009	0.014	−0.058**	−0.007	0.032**	−0.003
	(0.010)	(0.014)	(0.013)	(0.020)	(0.014)	(0.019)
Unskilled manual	−0.014	0.007	0.021	−0.017	0.024	−0.015
	(0.012)	(0.017)	(0.017)	(0.025)	(0.017)	(0.023)
**BIRTH SIZE (BASE CASE ***=*** VERY LARGE)**						
Larger than average	0.010**	−0.001	−0.010	−0.015	−0.017**	−0.008
	(0.005)	(0.007)	(0.007)	(0.011)	(0.007)	(0.010)
Average	0.016**	−0.005	−0.015**	−0.017	−0.015**	−0.017*
	(0.005)	(0.007)	(0.007)	(0.010)	(0.007)	(0.009)
Smaller than average	0.010*	−0.008	−0.010	−0.003	−0.020**	−0.008
	(0.005)	(0.008)	(0.008)	(0.012)	(0.008)	(0.011)
Very small	0.033**	−0.042**	−0.002	−0.032**	−0.034**	−0.041**
	(0.007)	(0.010)	(0.010)	(0.014)	(0.010)	(0.013)
Mother’s height	−0.000**	0.000**	−0.000	−0.000	0.000	0.000
	(0.000)	(0.000)	(0.000)	(0.000)	(0.000)	(0.000)
Mother’s weight	−0.000	−0.001**	0.001**	0.001**	0.000*	−0.000
	(0.000)	(0.000)	(0.000)	(0.000)	(0.000)	(0.000)
Agricultural household	−0.008**	0.002	−0.008	−0.005	0.003	0.004
	(0.004)	(0.006)	(0.006)	(0.008)	(0.006)	(0.008)
Total in household	−0.000	0.000	0.001**	0.004**	0.001**	0.002**
	(0.000)	(0.001)	(0.001)	(0.001)	(0.001)	(0.001)
Children in household	0.000	0.001	−0.007**	−0.002	−0.001	−0.014**
	(0.001)	(0.002)	(0.002)	(0.003)	(0.002)	(0.002)
Female	0.001	−0.004	−0.011**	−0.033**	0.004	0.007*
	(0.002)	(0.003)	(0.003)	(0.004)	(0.003)	(0.004)
Ever vaccinated	−0.012**	0.013**	0.007	0.030**	0.016**	0.031**
	(0.004)	(0.006)	(0.006)	(0.009)	(0.006)	(0.008)
Observations	29,630	29,630	29,630	29,630	29,630	29,630

*Standard errors (clustered on community-year) are given in parentheses*.

*All regressions include community-year fixed effects*.

*p < 0.10; **p < 0.05.

The results for infants between 1 and 2 years are presented in Table 4. Higher maternal education is associated with decreases in non-exclusive breastfeeding and increases in the use of infant formula, milk liquids, non-milk liquids, and solid foods. Maternal work status is correlated with decreases in the use of exclusive breastfeeding and infant formula and increases in the use of non-exclusive breastfeeding, milk liquids, and non-milk liquids. Mother’s weight is correlated with decreases in non-exclusive breastfeeding and solid foods and increases in the use of exclusive breastfeeding, infant formula, and milk liquids.

**Table 4:** **Associations with feeding type, age 1–2 years**.

Variable	Exclusive breastfeeding	Non-exclusive breastfeeding	Infant formula	Milk liquids	Non-milk liquids	Solids
**MOTHER’S EDUCATION (BASE CASE *=* NO FORMAL EDUCATION)**						
Incomplete primary	−0.001	−0.015**	0.013**	0.040**	0.002	0.003
	(0.002)	(0.006)	(0.003)	(0.005)	(0.003)	(0.003)
Complete primary	−0.002	−0.022**	0.016**	0.081**	0.009**	0.000
	(0.002)	(0.007)	(0.004)	(0.007)	(0.004)	(0.004)
Incomplete secondary	−0.001	−0.038**	0.026**	0.127**	0.003	0.008**
	(0.002)	(0.007)	(0.004)	(0.006)	(0.004)	(0.004)
Complete secondary	0.001	−0.090**	0.054**	0.158**	0.010*	0.012**
	(0.003)	(0.010)	(0.006)	(0.010)	(0.005)	(0.006)
Post-secondary	−0.005	−0.112**	0.098**	0.233**	0.022**	0.021**
	(0.005)	(0.014)	(0.008)	(0.014)	(0.007)	(0.008)
Mother is currently employed outside the home	−0.015**	0.041**	−0.017**	0.033**	0.011**	0.022**
	(0.004)	(0.010)	(0.006)	(0.010)	(0.005)	(0.006)
Mother works at home	−0.001	0.004	−0.006*	−0.001	0.001	−0.001
	(0.002)	(0.006)	(0.003)	(0.006)	(0.003)	(0.003)
**MOTHER’S OCCUPATION (BASE CASE ***=*** NO OCCUPATION)**						
Professional, technical, manager	0.008	−0.043**	0.065**	0.023	−0.003	−0.011
	(0.007)	(0.020)	(0.012)	(0.019)	(0.011)	(0.012)
Clerical	−0.004	−0.092**	0.080**	0.068**	0.021	0.012
	(0.009)	(0.026)	(0.015)	(0.025)	(0.014)	(0.015)
Sales	0.001	−0.053**	0.023**	−0.014	−0.000	0.003
	(0.005)	(0.015)	(0.009)	(0.015)	(0.008)	(0.009)
Agriculture-self-employed	−0.002	−0.018	0.036**	−0.024	0.005	0.006
	(0.006)	(0.016)	(0.009)	(0.016)	(0.009)	(0.009)
Agriculture-employe	−0.008	0.010	0.038**	−0.045**	0.013	0.014
	(0.006)	(0.017)	(0.010)	(0.017)	(0.009)	(0.010)
Domestic services	0.001	−0.076**	0.008	−0.036	0.022	0.019
	(0.009)	(0.026)	(0.015)	(0.026)	(0.014)	(0.015)
Services	0.017**	−0.067**	0.044**	0.032*	−0.025**	−0.002
	(0.007)	(0.020)	(0.011)	(0.019)	(0.010)	(0.011)
Skilled manual	−0.005	−0.019	0.028**	−0.032**	0.007	0.001
	(0.005)	(0.016)	(0.009)	(0.015)	(0.008)	(0.009)
Unskilled manual	−0.008	−0.029	0.008	−0.009	0.042**	0.012
	(0.007)	(0.020)	(0.012)	(0.020)	(0.011)	(0.012)
**BIRTH SIZE (BASE CASE ***=*** VERY LARGE)**						
Larger than average	0.002	0.011	−0.003	−0.024**	0.012**	0.000
	(0.003)	(0.009)	(0.005)	(0.009)	(0.005)	(0.005)
Average	0.001	0.025**	−0.003	−0.018**	0.010**	0.003
	(0.003)	(0.008)	(0.005)	(0.008)	(0.004)	(0.005)
Smaller than average	0.002	0.024**	−0.004	−0.009	0.005	0.004
	(0.003)	(0.009)	(0.005)	(0.009)	(0.005)	(0.005)
Very small	0.010**	0.011	−0.000	−0.012	−0.002	−0.010
	(0.004)	(0.011)	(0.007)	(0.011)	(0.006)	(0.007)
Mother’s height	−0.000	0.000	−0.000	0.000	0.000	0.001**
	(0.000)	(0.000)	(0.000)	(0.000)	(0.000)	(0.000)
Mother’s weight	0.000**	−0.003**	0.001**	0.001**	−0.000	−0.000**
	(0.000)	(0.000)	(0.000)	(0.000)	(0.000)	(0.000)
Agricultural household	0.013**	−0.011*	−0.012**	−0.034**	−0.005	−0.015**
	(0.002)	(0.007)	(0.004)	(0.007)	(0.004)	(0.004)
Total in household	−0.001**	0.004**	0.000	0.004**	0.001**	0.002**
	(0.000)	(0.001)	(0.000)	(0.001)	(0.000)	(0.000)
Children in household	0.011**	−0.001	−0.003**	−0.010**	−0.013**	−0.022**
	(0.001)	(0.002)	(0.001)	(0.002)	(0.001)	(0.001)
Female	0.003**	−0.008**	0.004*	−0.006*	−0.003	−0.009**
	(0.001)	(0.004)	(0.002)	(0.003)	(0.002)	(0.002)
Ever vaccinated	−0.011**	−0.027**	0.018**	0.021**	0.009**	0.034**
	(0.003)	(0.008)	(0.005)	(0.008)	(0.004)	(0.005)
Observations	52,780	52,780	52,780	52,780	52,780	52,780

*Standard errors (clustered on community-year) are given in parentheses*.

*All regressions include community-year fixed effects*.

*p < 0.1; **p < 0.05

## Discussion

Several mother, infant, and household factors are associated with the type of food given to an infant. Most interesting among these factors are those relating to the mother because she makes feeding decisions most frequently. A mother’s education, work status, and weight are all significantly correlated with the type of food given to the infant. We focus on these three factors in discussing the implications of this work. The significance of these factors is consistent with previous research and also accords with intuition. However, our analysis provides quantified estimates of their effect on food given to infants that can potentially be used in a general policy framework. We further discuss examples of ways in which these estimates can inform policymakers planning infant feeding programs.

Each of these three maternal factors has multiple pathways through which it may influence feeding decisions, and these pathways can act in opposite directions. For example, higher education increases health knowledge, which may lead to more exclusive breastfeeding in young infants and more appropriate complementary feeding in older infants. We control for work status in regressions, but education can also increase the frequency and intensity of work as well as the income of both the mother and her spouse (who likely has higher education as well). Both of these factors decrease the likelihood of exclusive breastfeeding by making time and opportunity costs more burdensome and increase the use of more expensive foods such as infant formula by increasing available financial resources (12).

Outlining these pathways is critical to understanding and interpreting the results of our analysis for policy planning purposes. As we see in this example, food types are affected differently: the increased opportunity costs of time associated with higher education (from increased wages) decreases the likelihood of exclusive breastfeeding but increases the likelihood of infant formula. Thus, by modeling these feeding patterns jointly, we are able to better gage their relative importance.

Our results show that the second effect dominates for the feeding of infants less than 6 months old because more infants of higher educated mothers are fed infant formula and milk liquids, although there is no consistent association with breastfeeding. The association with infant formula increases as education increases and the associations with both infant formula and milk liquids are relatively large. A mother with a secondary degree is 11 percentage points more likely to give her infant formula and 6 percentage points more likely to give her infant milk liquids than a woman with no formal education. These differences represent an 80% increase in mean infant formula use and a 33% increase in mean milk liquid use. Policymakers attempting to increase exclusive breastfeeding can use these differences to forecast the average impact that an infant feeding program aimed at high-educated, working mothers might have on exclusive breastfeeding (for example, policies to improve flexibility for mothers in high skilled workplaces). Based on these estimates, this type of program targeting may be a highly effective use of public health resources.

Our results also show that education’s combined effects of income and health knowledge operate in the same direction for infants aged 6 months or older. This is likely because complementary feeding is recommended to be introduced in this age range. These effects are also relatively large. A mother with a secondary degree is 18 percentage points more likely to give her infant formula and 11.5 percentage points more likely to give her infant milk liquids than a woman with no formal education. These changes represent a 105% increase in mean infant formula use and a 33% increase in mean milk liquid use. These parameters enable policymakers to forecast the average impact of complementary feeding education programs targeting low educated mothers of older infants for whom complementary feeding is appropriate. Based on these estimates, this type of program targeting may be a highly effective use of public health resources.

A mother’s working status is another important indicator of the time and financial resources available for feeding her infant. Mothers that work have less time to spend breastfeeding and more money to spend on other foods. It is important to emphasize that we control for education and occupation, which are correlated with the likelihood of working and wages. For infants younger than 6 months, our results show that working is associated with a decrease in exclusive breastfeeding and that this decrease is made up for by an increase in non-exclusive breastfeeding. We also find a positive relationship in the use of milk liquids, non-milk liquids, and solid foods. These associations are relatively large representing a 22% decrease from the mean level of exclusive breastfeeding and 17, 12, and 30% increases in the mean levels of milk liquids, non-milk liquids, and solid foods, respectively. Interestingly, a mother’s working status is not correlated with the use of infant formula. Conversely, for infants aged 6 months to 1 year, work status is associated with increased usage of infant formula. This represents 18% of mean infant formula use. It may be that mothers prefer infant formula only for these older infants. Finally, for infants aged 1–2 years, the association of a mother working with all other foods (except infant formula) is strong and positive; the association is negative for infant formula, which may reflect the increasing introduction of complementary foods. Policymakers planning programs to increase the use of appropriate complementary food in older infants (age 1–2 years) can use these estimates to forecast the average impact of a program targeting non-working mothers with resources for complementary foods. Based on these estimates, this type of program targeting may be a highly effective use of public health resources.

In the settings examined here, higher maternal weight indicates improved health, and this can affect feeding patterns in two ways. First, better health increases a woman’s ability to breastfeed. Second, a healthier woman is better able to work both outside and inside the home at household production, which takes away time from breastfeeding and increases household resources available for more expensive foods, such as infant formula and milk liquids. We find that both of these factors are at play with heavier women more likely to exclusively breastfeed and more likely to give their infants infant formula and milk liquids. However, the magnitude of these associations is relatively small. For example a 5 kg increase in maternal weight will only increase exclusive breastfeeding by 1.5% from the mean level. Policymakers can use these results to forecast the average impact of programs to increase breastfeeding by improving maternal health. In this case, these estimates indicate that program targeting of this sort may not be as effective at improving infant feeding as other potential programs.

Our study has several limitations. First, although we have indicators for multiple feeding behaviors, we lack important data on the intensity of each type of feeding. Second, our data consist of multiple cross-sections, but we lack the ability to track households across time. Longitudinal data would allow for more precise estimation of the correlates of infant feeding. This analysis attempts to provide unbiased estimates of the association between feeding practices and maternal, household, and infant factors by controlling for community-year fixed effects and all of the reported independent variables. This approach arguably controls for much of the potential bias from confounding factors (as discussed above), but household or infant-specific factors not controlled for by the included variables could continue to bias estimates. We also employed the 24-h dietary recall, which may provide some measurement error since feeding behaviors were not tracked over a longer period of time. Thus, it is possible that the infant occasionally receives some other foods but simply did not the previous day which would result in an overestimate of the prevalence of exclusive breastfeeding (13).

This study provides quantified estimates of the associates between maternal, household, and infant factors and diverse infant feeding practices. By quantifying associations that have in the past been based in preliminary research and intuitive logic, we provide important information for public health policymakers seeking to motivate improvement in infant feeding. The generalized population averages that are produced in this research are necessary when forecasting the average impact of population policies, because they provide policymakers across a broad spectrum with average associations between household, maternal, and child factors and infant feeding practices. They will help policymakers determine which targeted programs will on average have the greatest impact.

## Conflict of Interest Statement

This research was funded by Nestle Nutrition Institute. The funding organization is an independent institute that is associated with a company that produces infant formula. Patrick Detzel is an employe of Nestlé Research Center.

## Appendix

**Table A1:** **Countries and years in sample**.

Country	Years
Bangladesh	1999
Benin	2006, 2001, 1996
Bolivia	2003, 1998
Cameroon	2004, 1998, 1991
Colombia	2010, 2005, 2000, 1995
Dominican republic	2007, 1999, 1996
Egypt	2008, 2005, 2000, 1996
Ghana	2008, 2003, 1993
India	2005, 1992
Indonesia	2007
Kenya	2008, 2003, 1998, 1993
Madagascar	2008, 2003, 1997, 1992
Mali	2001, 1995
Nepal	2006, 1996
Philippines	2008, 1998, 1993
Senegal	2005
Tanzania	2004, 1999, 1996
Turkey	1998, 1993
Uganda	2006, 2000, 1995
Zambia	2007, 2001, 1996, 1992

**Table A2:** **Food type definitions**.

Infant formula	Milk liquids	Non-milk liquids	Solid foods
Commercially produced baby formula	Powdered or tinned milk Fresh milk	Plain water Sugar water Juice Tea or coffee	Baby cereal porridge/gruel Bread, noodles, other foods made from grains
			Potatoes, cassava or other tubers
			Eggs
			Meat
			Pumpkin, carrots, squash
			Any dark green leafy vegetables
			Mangoes, papayas, other vitamin A fruits
			Any other fruits
			Liver, heart, other organs
			Fish or shellfish
			Food made from beans, peas, lentils, nuts
			Cheese, yogurt, other milk products
			Oil, fats, butter
			Chocolates, sweets, candies, pastries
			Eggs, fish, poultry
			Any other solid or semi-solid foods
			Food made from wheat, maize, rice, sorghum, or other local grains
